# Unpleasant meditation-related experiences in regular meditators: Prevalence, predictors, and conceptual considerations

**DOI:** 10.1371/journal.pone.0216643

**Published:** 2019-05-09

**Authors:** Marco Schlosser, Terje Sparby, Sebastjan Vörös, Rebecca Jones, Natalie L. Marchant

**Affiliations:** 1 Division of Psychiatry, Faculty of Brain Sciences, University College London, London, United Kingdom; 2 Department of Psychology and Psychotherapy, Faculty of Health, Witten/Herdecke University, Witten, Germany; 3 Department of Philosophy, Faculty of Arts, University of Ljubljana, Ljubljana, Slovenia; Bangor University, UNITED KINGDOM

## Abstract

So far, the large and expanding body of research on meditation has mostly focussed on the putative benefits of meditation on health and well-being. However, a growing number of reports indicate that psychologically unpleasant experiences can occur in the context of meditation practice. Very little is known about the prevalence and potential causes of these experiences. The aim of this study was to report the prevalence of particularly unpleasant meditation-related experiences in a large international sample of regular meditators, and to explore the association of these experiences with demographic characteristics, meditation practice, repetitive negative thinking, mindfulness, and self-compassion. Using a cross-sectional online survey, 1,232 regular meditators with at least two months of meditation experience (mean age = 44.8 years ± 13.8, 53.6% female) responded to one question about particularly unpleasant meditation-related experiences. A total of 315 participants (25.6%, 95% CI: 23.1 to 28.0) reported having had particularly unpleasant meditation-related experiences, which they thought may have been caused by their meditation practice. Logistic regression models indicated that unpleasant meditation-related experiences were less likely to occur in female participants and religious participants. Participants with higher levels of repetitive negative thinking, those who only engaged in deconstructive types of meditation (e.g., vipassana/insight meditation), and those who had attended a meditation retreat at any point in their life were more likely to report unpleasant meditation-related experiences. The high prevalence of particularly unpleasant meditation-related experiences reported here points to the importance of expanding the scientific conception of meditation beyond that of a (mental) health-promoting and self-regulating technique. We propose that understanding when these experiences are constitutive elements of meditative practice rather than merely negative effects could advance the field and, to that end, we conclude with an overview of methodological and conceptual considerations that could be used to inform future research.

## Introduction

Meditation practices and their psychological and neurobiological effects have been studied extensively [1]. Much of the research output of the nascent field of contemplative science has focussed on the beneficial aspects of meditation [2]. This narrow investigative scope has implicitly and explicitly constructed an image of meditation as a panacea for an ever-increasing host of psycho-physical ailments. However, until recently, contemporary research has paid little attention to *particularly unpleasant* meditation-related experiences (e.g., anxiety, fear, distorted emotions or thoughts). In fact, a careful study of the literature reveals that most studies on meditation have not explored experiences that meditators would describe as particularly unpleasant or difficult [3]. Some researchers have recently tried to remedy this investigative lacuna by expanding the range and diversity of meditative experiences that fall within the ambit of empirical science [4, 5].

However, from a clinical and scientific perspective, it is still largely unclear whether, and how, meditation is implicated in particularly unpleasant meditation-related experiences and how to properly measure, investigate, or even define them. The discussion of particularly unpleasant meditation-related experiences through the lens of traditional contemplative traditions is similarly opaque. Traditional Buddhist textual sources indeed contain vivid accounts of particularly unpleasant meditation-related experiences and elaborate interpretative frameworks to help meditators understand them; yet these accounts vary widely, are couched in tradition-specific terms, and often revert to polemic and prescription [6–8]. Consequently, no single authoritative Buddhist account of what constitutes particularly unpleasant meditation-related experiences can be straightforwardly extracted from historical sources to be conveniently operationalised in contemporary empirical research. For example, whether particularly unpleasant meditation-related experiences are framed as inherent stages of the contemplative path and even markers of progress towards liberation, or whether they are viewed as avoidable hindrances caused by, for instance, excessive striving, can differ between and within Buddhist traditions. These are just two examples amongst a myriad of traditional meaning-making narratives (for further discussion see [9]). Further complicating matters is the fact that an open and mutually enriching discussion between Buddhist, scientific, and clinical camps around particularly unpleasant meditation-related experiences has not yet occurred. However, models for how such an interdisciplinary and cross-cultural dialog can be encouraged have recently been proposed [10, 11].

The small body of literature on meditation-related difficulties and extreme states associated with meditation consists mostly of case reports and case series that have linked meditative practices with instances of anxiety, panic, depersonalisation, mania, psychosis, suicidality, and an exacerbation of clinical symptoms [12–27]. Notwithstanding their importance for illuminating unchartered scientific ground, case reports do not lend themselves to clearly disentangling the causal or catalysing effects of meditation from prodromal or latent mental health problems [28]. The only prospective study on difficult meditation-related experiences to date indicated that 2 (7.2%) of the 27 participants that attended a vipassana meditation retreat reported profound adverse effects that led to the discontinuation of their practice [29].

In the first larger-scale cross-sectional study on unwanted meditation-related experiences, 342 meditators with at least two months of meditation experience were asked if they had “experienced any type of unwanted (not normally expected) or adverse reactions (with potential harm to their health) resulting from [the] practice of meditation” [4]. A total of 25.4% of participants reported unwanted effects. Most effects were transitory, did not require the interruption of the meditation practice, were more likely to occur when practicing alone rather than in a group context, and were positively associated with the length of the meditation session. As part of a recent survey [30] developed by a task force of meditation researchers and teachers on neglected yet important topics in meditation research (e.g., mystical and anomalous experiences), 1,120 meditators– 71% of which had maintained a regular practice over the past 6 months—were asked one question about difficult meditation-related experiences. From the total sample, 32% of meditators indicated that they had “experienced disturbing feelings of fear, dread, or terror” during or just after meditating. A recent mixed-methods study on practice-related challenges recruited 60 experienced Western Buddhist meditators and meditation teachers across traditions who had experienced distress in the context of their meditation practice [5]. Almost half of practitioners (45%) were first confronted with meditation-related difficulties between 1 and 10 years of meditation, while a smaller subset of practitioners (12%) encountered challenges within 10 days of starting a meditation practice. Qualitative analysis identified four domains of influencing contextual factors for challenges: practice-related (e.g., intensity, amount, type), meditator/practitioner-related (e.g., personality, interpretive framework, medical history, intentions), relationship-related (e.g., meditation community, sociocultural context, practice environment), and health behaviour-related factors (e.g., diet, sleep, exercise, psychotherapy, drugs). Additionally, the authors investigated causal attribution to meditation using pre-specified causality assessment criteria (e.g., subjective attribution, temporal proximity). The variety of contemplative experiences spanned seven phenomenological domains (e.g., cognitive, perceptual, affective) and included fear, anxiety, hallucinations, social impairment, and changes in motivation, worldviews, self-world boundaries, and sleep. In another qualitative study of 30 male meditators, most of whom were recruited from a single Buddhist community in London, the authors reported that one quarter of their data comprised accounts of experiential challenges that occurred during meditation [31]. Meditation-related challenges included the exacerbation of mental health problems, distressing perceptual experiences of the sense of self, and one case of hospitalisation due to a psychotic episode.

Related research on mindfulness-based interventions has generally not included measurement or evaluation of potential meditation-related difficulties. A recent systematic review of randomised controlled trials of mindfulness-based stress reduction and mindfulness-based cognitive therapy found that only 1% of the 4031 participants included in 36 randomised controlled trials reported “adverse events” [32]. Importantly, the review indicated that 15.6% (36 out of 231) of eligible trials contained an adverse events statement in their protocol. In comparison, 21% of psychological intervention trials (published during the calendar year of 2010) [33] and 100% of pharmacological trials (published in high-impact journals between 1992 and 2009) monitored adverse events [34]. This review corroborated earlier findings indicating that fewer than 25% of meditation trials included methods for assessing adverse events [3, 33]. Meditation-related difficulties are therefore likely to be underreported. No specific clinical guidelines clearly addressing practice-related adverse events have been published, however, efforts have been made to ameliorate this situation (see 1). For example, recent protocols on mindfulness-based stress reduction and mindfulness-based cognitive therapy mention potential risks to participants [35, 36].

Empirical evidence regarding the relationship between particularly unpleasant meditation-related experiences and meditation types is sparse. A recently proposed taxonomy grouped meditation practices into attentional, constructive, and deconstructive practices based on their underlying cognitive mechanisms [37]. Briefly, attentional meditation types primarily train the ability to sustain attention on phenomena without becoming absorbed by them (e.g., mindfulness of breathing), and constructive types primarily nurture cognitive and emotional patterns conducive to well-being (e.g., loving-kindness meditation). Deconstructive practices aim to weaken and dissolve the implicit belief in the inherent and independent existence of objects of consciousness including conceptual frameworks, views and models of the self and others, the world and of consciousness itself (e.g., vipassana/insight meditation). When considering these definitions to generate exploratory hypotheses for the present study, difficult and particularly unpleasant experiences could be expected to occur more frequently in the context of deconstructive practices than attentional and/or constructive practices.

In brief, research on meditation and mindfulness-based interventions seldom include measures of particularly unpleasant meditation-related experiences, and standardised assessment methods for such experiences have not yet been developed and implemented. Subsequently, prevalence estimates of particularly unpleasant meditation-related experiences across secular and contemplative contexts remain inconclusive, while preliminary findings suggest that their occurrence is highly dependent on a complex interaction of contextual factors. In the context of 18 million meditators in the United States alone [38, 39, 40], even low rates of particularly unpleasant meditation-related experiences—comparable, for instance, to the rate of severe side effects during psychotherapy (5%) [41, 42]–become an important concern not only for the nascent field of contemplative science but for public health more generally. The aim of the present study was to report the prevalence of particularly unpleasant meditation-related experiences in a large international sample of regular meditators and, based on previous research [4, 5, 30], to explore the relationship of these experiences with demographic characteristics, meditation practice, repetitive negative thinking, mindfulness, and self-compassion. This article concludes with an outline of methodological and conceptual considerations that could inform future research on particularly unpleasant meditation-related experiences. We propose that an interdisciplinary discussion could enable research approaches that aim to investigate the potential distinction between negative experiences and experiences that are difficult, but essential for the wider benefits of meditative training to unfold.

## Method

Individuals were informed that the study aimed to advance the scientific understanding of meditation practices and their relationship to cognitive and emotional processes, and that by submitting their completed questionnaire they consented to participate. This study and consent procedure was approved by the University College London ethics committee (ref no: 10043/001) and conducted in line with the Declaration of Helsinki. This study used a cross-sectional design.

### Participants and data collection

An anonymous online survey that took approximately 20 minutes to complete was used between April and August 2017 to recruit regular meditators (practicing at least once a week) with a minimum age of 18 years and a good understanding of the English language. The survey link was shared on social media (Facebook, Twitter) and sent to Buddhist communities, meditation centres, and mindfulness associations. A total of 2,599 individuals started the survey, and 1,706 participants completed it. We excluded a total of 474 participants: 383 indicated only a yoga practice; 42 did not indicate a meditation practice; 34 had less than two months of meditation experience; another 15 did not respond to the question about particularly unpleasant meditation-related experiences and were also excluded. Therefore, 1,232 participants were included in the subsequent analyses. Individuals did not receive a financial reward for participation.

### Measures

Participants were asked to report demographic information and to answer the following question regarding particularly unpleasant meditation-related experiences: “Have you ever had any particularly unpleasant experiences (e.g., anxiety, fear, distorted emotions or thoughts, altered sense of self or the world), which you think may have been caused by your meditation practice?”. The term “particularly unpleasant” was chosen over other potential labels (e.g., adverse, aberrant, negative, challenging, unwanted), which we evaluated as more likely to predetermine the value and significance (or lack thereof) of the experience and nudge participants towards theorising about their experiences. Meditators also reported their total years of regular practice (lifetime meditation experience), weekly number of meditation sessions (frequency), average length of their meditation sessions (session length), whether they had ever attended a meditation retreat (retreat experience), and the types of meditation they practiced. Meditation types were based on a taxonomy categorising meditation practices into attentional, constructive, and deconstructive types [36]. The following practices were categorised as attentional meditation types: mindfulness of breathing, breath counting, Jhana practice, samatha/samadhi practice, visualisation, mantra recitation, Kirtan Kriya, choiceless awareness, and mindfulness meditation (e.g., as taught by mindfulness-based stress reduction programmes). Loving-kindness and compassion meditation were listed as constructive types. Vipassana/insight meditation, Mahamudra, Dzogchen, Shikantaza/‘just sitting’, Self-inquiry, and Koan practice were listed as deconstructive types (S1 Appendix).

Repetitive negative thinking was measured using the Perseverative Thinking Questionnaire (PTQ) [43]. The PTQ is a 15-item measure that uses a 5-point Likert scale ranging from 0 (never) to 4 (almost always) to capture levels of repetitive negative thinking.

Participants were asked to indicate how they *typically* think about negative experiences or problems. Total PTQ scores are computed by summing all item scores (possible range: 0 to 60), with higher scores indicating higher levels of repetitive negative thinking. PTQ scores are associated with symptoms of anxiety and depression, and the PTQ has displayed excellent psychometric properties across samples: Cronbach’s alpha ranged from 0.94 to 0.95 [43].

Self-compassion was measured using the Self-Compassion Scale (SCS) [44]. The SCS is a 26-item scale that uses a 5-point Likert scale ranging from 1 (almost never) to 5 (almost always) to capture levels of self-compassion. Participants were asked to indicate how they *typically* act towards themselves in difficult times. The total self-compassion score (possible range: 1 to 5) is obtained by computing the grand mean of the positive subscales (self-kindness, common humanity, and mindfulness) and the reverse scored negative subscales (self-judgment, isolation, and over-identification). Higher total scores indicate higher levels of self-compassion. The SCS has displayed good psychometric properties across samples: Cronbach’s alpha for the total scale ranged from 0.91 to 0.95 [45].

Mindfulness was measured using the Mindsens composite index [46], a 19-item measure comprising items from the Five Facet Mindfulness Questionnaire (FFMQ) [47] and the Experience Questionnaire (EQ) [48]. The Mindsens was derived from a factor analysis that included all items from the FFMQ and EQ and three meditation-related variables (total months, days per month, and average session length of meditation practice). Using the Mindsens (Cronbach’s alpha = 0.91), non-meditators could be discriminated from meditators in 82% of cases [44]. A 5-point Likert scale ranging from 1 (never or very rarely true) to 5 (very often or always true) assesses capacity to be mindful in daily life and to observe mental events as transient. Participants were asked to indicate what is *generally* true for them. A total score (possible range: 1 to 5) is obtained by averaging all 19 item scores.

### Statistical analyses

To assess the association between particularly unpleasant meditation-related experiences and demographic variables, repetitive negative thinking, mindfulness, and self-compassion, we fitted exploratory univariable logistic regression models that included particularly unpleasant meditation-related experiences (yes vs no) as the binary outcome variable and demographic variables, repetitive negative thinking, mindfulness, and self-compassion as the explanatory variables (binary: sex, education, religiosity; continuous: age, mindfulness, self-compassion, repetitive negative thinking). An adjusted multivariable model with continent of residence as a covariate was fitted to adjust for possible confounding of the association between religiosity and particularly unpleasant meditation-related experiences. A binary variable to denote deconstructive and non-deconstructive meditation types was generated (meditation type). The deconstructive group (n *=* 176) included meditators who only engaged in deconstructive types, whereas the non-deconstructive group (n *=* 557) included meditators who only used attentional and/or constructive types. Meditators who used both deconstructive and non-deconstructive types of meditation were not considered, as this mixed group would not differentiate participants who regularly engage in both types of meditation from those who almost exclusively engage in either a deconstructive or a non-deconstructive type; hence, this category would be conceptually too broad to offer meaningful findings. We then fitted univariable logistic regression models to explore the association between particularly unpleasant meditation-related experiences and meditation practice variables (continuous: lifetime meditation experience, frequency, session length; binary: retreat experience, meditation type). Stata/IC 13.0 (Stata Corp, College Station, TX) was used for all analyses.

## Results

Participant characteristics are displayed in Table 1. Of the 1,232 participants, 25.6% (n = 315, 95% CI: 23.1 to 28.0) indicated that they had previously encountered particularly unpleasant meditation-related experiences, which they thought may have been caused by their meditation practice. Approximately half of meditators were female (n = 660, 53.6%), and most were religious (n = 756, 61.4%) and had received a university degree (n = 899, 73.0%). Participants had an average age of 44.8 years ± 13.8 (range 18–82) had, on average, sustained a regular mediation practice for a median of 6 years (inter-quartile range: 2.5 to 13.0) during which they meditated on average 10.3 times per week ± 6.6 with a mean session length of 28 minutes ± 16. The majority of participants had attended at least one meditation retreat (n = 782, 63.5%).

**Table 1:** Demographic and meditation-related characteristics of 1,232 regular meditators.

	Missing values—n (%)	Summary statistic ^a^
Age (years)–mean (SD)	0	44.8 (13.8)
Sex	6 (0.5%)	
Female		660 (53.6%)
Education	18 (1.5%)	
Completed a university degree		899 (73.0%)
Religion	31 (2.5%)	
Religious		756 (61.4%)
Continent of residence	35 (2.8%)	
Europe		447 (36.3%)
Asia		372 (30.2%)
North America		283 (23.0%)
Australia and New Zealand		73 (5.9%)
South America		15 (1.2%)
Africa		7 (0.6%)
Meditation practice variables	0	
Particularly unpleasant experiences		315 (25.6%)
Meditation experience (years)–median (IQR)		6 (2.5 to 13.0)
Starting age (years)–mean (SD)		34.8 (12.5)
Session frequency (per week)–mean (SD)		10.3 (6.6)
Session length (minutes)–mean (SD)		28 (16)
Retreat experience (at any point in life)		782 (63.5%)
Meditation types ^b^	0	
Attentional		1,010 (82.0%)
Deconstructive		675 (54.8%)
Constructive		435 (35.3%)
Repetitive negative thinking—mean (SD)	0	22.5 (9.5)
Mindfulness—mean (SD)	0	3.7 (0.7)
Self-compassion—mean (SD)	0	3.6 (0.6)

Note: SD = standard deviation; IQR = inter-quartile range

^**a**^Statistics in this column are n (%) unless otherwise specified.

^b^The total percentage exceeds 100% as 48.2% of meditators practiced more than one type of meditation.

We found strong evidence that religious participants have lower odds of having particularly unpleasant meditation-related experiences (odds ratio = 0.64; 95% CI: 0.49 to 0.83; *p* = 0.001). This association was only slightly attenuated after adjustment for continent of residence (adjusted OR = 0.72; 95% CI: 0.54 to 0.96; *p* = 0.024). We found weak evidence that female participants were less likely to have unpleasant meditation-related experiences (OR = 0.75; 95% CI: 0.58 to 0.97; *p* = 0.030) and for an association between higher levels of repetitive negative thinking and unpleasant meditation-related experiences (OR = 1.02, 95% CI: 1.00 to 1.03, *p* = 0.025). Regarding meditation practice variables, we found evidence that the odds of particularly unpleasant meditation-related experiences were 65% higher in meditators who only engaged in deconstructive practices compared to meditators who only engaged in non-deconstructive practices (OR = 1.65; 95% CI: 1.12 to 2.42; *p* = 0.011). Although unpleasant experiences could not be conclusively linked to the time of retreat, we found strong evidence for an association between particularly unpleasant meditation-related experiences and retreat experience (OR = 1.68; 95% CI: 1.27 to 2.23; *p* < 0.001). We found no evidence for an association between particularly unpleasant meditation-related experiences and lifetime meditation experience (OR = 1.00; 95% CI: 0.99 to 1.01; *p* = 0.738), frequency (OR = 1.01; 95% CI: 0.99 to 1.03; *p* = 0.290), or session length (OR = 1.06; 95% CI: 0.98 to 1.15; *p* = 0.134). Table 2 displays all logistic regression models. Assumptions were met for all logistic regression models.

**Table 2:** Associations with particularly unpleasant meditation-related experiences.

	Unpleasant experiences			
Binary predictors	% (n)^a^	Odds ratio^b^	95% CI	*p*-value
Age (1 year)	–	0.99	0.98 to 1.00	0.081
Sex				
Male	28.5% (161)	–	–	–
Female (vs male)	23.0% (152)	0.75	0.58 to 0.97	0.030
Education				
Did not receive university degree	22.5% (71)	–	–	–
Received university degree (vs did not)	26.7% (240)	1.25	0.92 to 1.69	0.146
Religiosity				
Not religious	30.6% (136)	–	–	–
Religious (vs not religious)	22.0% (166)	0.64	0.49 to 0.83	0.001
Lifetime meditation experience (1 year)	–	1.00	0.99 to 1.01	0.738
Meditation type				
Non-deconstructive	20.3% (113)	–	–	–
Deconstructive only (vs non-deconstructive)	29.6% (52)	1.65	1.12 to 2.42	0.011
Retreat experience (at any point in life)				
No experience	19.6% (88)	–	–	–
Experience (vs no experience)	29.0% (227)	1.68	1.27 to 2.23	<0.001
Frequency (per week)	–	1.01	0.99 to 1.03	0.290
Session length (10 minutes)	–	1.06	0.98 to 1.15	0.134
Repetitive negative thinking (1 SD)	–	1.02	1.00 to 1.03	0.025
Mindfulness (1 SD)	–	1.02	0.84 to 1.24	0.835
Self-compassion (1 SD)	–	0.93	0.76 to 1.15	0.516

Note: CI = confidence interval; SD = standard deviation

^a^No summary statistics are presented for continuous predictors.

^b^For binary explanatory variables (sex, education, religiosity, meditation type, retreat experience), the estimate describes the odds of particularly unpleasant meditation-related experiences in one group relative to the reference category (indicated in parentheses). For continuous explanatory variables (age, lifetime meditation experience, frequency, session length, repetitive negative thinking, mindfulness, self-compassion) the estimate reflects the expected increase in the odds of particularly unpleasant meditation-related experiences for a one unit increase in the explanatory variable.

## Discussion

Surveying over one thousand regular meditators, this is the largest cross-sectional study to assess particularly unpleasant meditation-related experiences to date. Approximately one quarter of participants reported that they had encountered particularly unpleasant meditation-related experiences (e.g., anxiety, fear, distorted emotions or thoughts, altered sense of self or the world) in the past. To our knowledge, only two other large quantitative studies of unpleasant meditation-related experiences have been published. In the first study (n = 342) [4], 25.4% of meditators with at least two months of meditation experience reported “unwanted effects”. In the second study (n = 1,120) [30], 32% of meditators reported “fear, dread, or terror” during or shortly after meditating. The high prevalence reported here and previously points to the importance of expanding the scientific conception of meditation beyond that of a (mental) health-promoting, stress-reducing, attention-enhancing, self-regulating technique.

However, several important points should be kept in mind when considering the present findings. First, in the present study only one question was used to capture the prevalence of particularly unpleasant meditation-related experiences. Our data did not provide any indication of the exact type of experiences or their severity and impact. Second, how data regarding particularly unpleasant meditation-related experiences are collected is likely to affect the rate of reporting. For instance, although actively prompting meditators to disclose potential particularly unpleasant meditation-related experiences might increase the rate of reporting compared to passive monitoring, medical research indicates that the estimates from active monitoring might be more accurate [49, 50]. Third, recent interest evaluating particularly unpleasant meditation-related experiences in meditation research and the absence of standardised measures have likely led to an *underestimation of the actual rate* of particularly unpleasant meditation-related experiences, coupled with an *exaggeration of their intensity*, given that only extreme cases would have come to the attention of researchers and clinicians. In other words, passive monitoring in meditation research could have led to an underreporting of particularly unpleasant meditation-related experiences. This proposal is reflected in the published literature containing a host of case reports of severe meditation-related experiences, yet not including any long-term randomised controlled trials of regular meditation that have quantitatively or qualitatively captured particularly unpleasant meditation-related experiences. Fourth, we did not assess possible pre-existing mental health problems, which could have confounded our prevalence estimate of particularly unpleasant meditation-related experiences. The reported prevalence estimate could therefore be more a reflection of the lifetime or 12-month prevalence of mental health problems in the general population [51, 52]. Relatedly, participants with high levels of anxiety and depression, for instance, could be more likely to maintain a regular meditation practice to manage their symptoms, which, without the skilful guidance of an experienced meditation teacher, could increase the likelihood of particularly unpleasant meditation-related experiences [53]. Nonetheless, it should be highlighted that the assessment of unpleasant meditation-related experiences in the present study included a form of subjective causality attribution, which suggests that the reported prevalence estimates cannot be entirely explained by citing base rates of mental health problems in the general population. In brief, longitudinal controlled studies, informed by clear definitions of particularly unpleasant meditation-related experiences, are needed in order to further our understanding of the prevalence, nature, and impact of unpleasant phenomena that can be encountered as a result of meditation.

Interestingly, religious participants were less likely to report particularly unpleasant meditation-related experiences. The research literature on religiosity and mental health offers two entangled empirical narratives: some studies suggest that religious beliefs can present a psychological buffer against stress and hopelessness; other studies, however, indicate an intricate link between religious beliefs and psychotic and neurotic disorders [54–56]. Religious frameworks can, at times, cause and exacerbate mental health problems when individuals are confronted with emotional challenges [57]. As mentioned above, previous research suggested that the way the phenomenology is interpreted depends, at least in part, on the meditator’s worldview and frameworks. Interestingly, the presence of several conflicting interpretive frameworks (e.g., from Buddhism and psychiatry) operating within a given practice context might encumber a practitioner’s ability to appraise unpleasant meditation-related experiences in helpful ways [5, 22]. One might surmise that meditators who explicitly identify as religious are potentially more likely to be embedded in one comprehensive, and (at least seemingly) internally consistent worldview, rather than several partially contradicting worldviews. This perceived consistency could serve as a resource for preventing or reducing the occurrence of particularly unpleasant meditation-related experiences. Further, the role of ethics in religious and contemplative traditions is commonly conceptualised as indispensable on the path of long-term meditation training. Relatedly, the potential buffering of religiosity on particularly unpleasant meditation-related experiences could be due to a stronger sense of community, more regular contact with fellow practitioners, and easier access to qualified meditation teachers and their guidance. Another explanation for our findings could be that religious meditators are simply less likely to report particularly unpleasant meditation-related experiences even if they occurred, because their beliefs about mental health problems (including severely unpleasant meditation-related experiences) may be imbued with stigma [58, 59]. Similarly, particularly unpleasant meditation-related experiences could threaten an individual’s religious or cultural identity, and could even cause them to leave their religion.

Male compared to female meditators had a higher likelihood of reporting particularly unpleasant meditation-related experiences. Lomas et al. (2014) [31] proposed that the emotion regulation paradigm [60] is a helpful theoretical framework for understanding potential sex differences in experiencing, expressing, and processing difficult and distressing emotions in the context of meditation practice. Literature suggests that men experience more restrictive emotionality and emotion regulation difficulties [61, 62], which may provide a useful framework for generating hypotheses for future studies on particularly unpleasant meditation-related experiences. Relatedly, several studies indicated that men benefited less from meditation training than women [63, 64]. Overall, more research is needed to understand why men would be more likely to report particularly unpleasant meditation-related experiences despite a prominent sex difference in affective disorders (i.e., anxiety and depression) observed in clinical research (for a review see [65]). Given the cross-sectional nature of our data and the weak evidence for sex differences in particularly unpleasant meditation-related experiences observed here, we caution against extrapolating strong conclusions without further research.

Participants with higher levels of repetitive negative thinking were more likely to report particularly unpleasant meditation-related experiences, although the estimate for this association was small in magnitude. Repetitive negative thinking is a transdiagnostic process comprising worry and rumination. Heightened levels of repetitive negative thinking are most notably implicated in the maintenance and development of depressive and anxiety disorders but also manifest across a wide range of other mental health problems, including psychosis, insomnia, and post-traumatic stress disorder [66]. Our data precludes any causality assessment between repetitive negative thinking and particularly unpleasant meditation-related experiences. It could be that meditators predisposed to heightened levels of repetitive negative thinking may be more susceptible to particularly unpleasant meditation-related experiences because they lack the ability to disengage from intrusive and repetitive negative content that arises during meditation. An alternative explanation is that the very occurrence of particularly unpleasant meditation-related experiences could trigger prolonged periods of repetitive negative thinking.

Regarding the relationship between meditation practice variables and particularly unpleasant meditation-related experiences, it should be noted that we only asked participants about the general characteristics of their meditation practice and did not collect detailed information pertaining to the characteristic of the practice periods during which these experiences occurred. Keeping this caveat in mind, our findings indicate that meditators who only engaged in deconstructive types of mediation (e.g., vipassana/insight meditation) were more likely to report particularly unpleasant meditation-related experiences than meditators who only engaged in attentional (e.g., mindfulness of breathing) and/or constructive types (e.g., loving-kindness meditation). In some contemplative traditions, attentional and constructive meditation types are occasionally conceptualised as practices that can cognitively and emotionally prepare the meditator—for example, by strengthening meta-awareness and nurturing acceptance—for the difficult experiences that can arise within the context of intensive deconstructive meditation practice [37]. In deconstructive practices, the direct phenomenological inquiry into the emotional, cognitive, and perceptual patterns and dynamics of lived experience can fundamentally challenge our usual ways of seeing and, thus, be associated with particularly unpleasant meditation-related experiences. For instance, insight meditation practices often encourage meditators to attune their attention to the impermanent, unsatisfactory, and impersonal nature of thoughts, feelings, and body sensations that arise within the space of awareness. Perceiving phenomena that might commonly be conceived of as inherently permanent and separate (e.g., the sense of self) as a vibrating field of fleeting and interpenetrating sensations could, for instance, give rise to a fear of annihilation [67]. Another potential interpretation of this relationship is that meditators prone to particularly unpleasant meditation-related experiences are more likely to engage in deconstructive practices because they seek to investigate and weaken the maladaptive cognitive patterns that might trigger and perpetuate particularly unpleasant meditation-related experiences.

Meditators with retreat experience were more likely to report particularly unpleasant meditation-related experiences. Importantly, our data does not capture whether the particularly unpleasant meditation-related experiences happened before, during, or after a meditation retreat, or whether and how these experiences may have been linked to the unique constellation of influencing factors operating in the context of retreat practice [68]. Retreat experience could simply be a proxy for other variables that underlie the relationship to particularly unpleasant meditation-related experiences (e.g., intentions, personality). Alternatively, meditators more prone to particularly unpleasant meditation-related experiences may be more likely to attend retreats as most retreat environments offer a direct and individualised supervision by experienced teachers. Previous studies on unpleasant meditation-related experiences that measured contextual practice-related factors found associations with meditation retreats. For instance, Lindahl et al. (2017) [5] reported that three-quarters of meditation-related difficulties were encountered during or immediately after a meditation retreat. In slight contrast, Cebolla et al. (2017) [4] suggested that group practices or contexts in which meditators feel more connected to fellow practitioners could potentially have a buffering effect. For the benefit of future research exploring specific associations between retreats and particularly unpleasant meditation-related experiences, we believe it is valuable to briefly highlight how retreat-specific characteristics could be delineated [5]: (i) practice-related: practice intensity is high, with long and frequent sessions; (ii) relationship-related: commonly, retreats are held in silence, take place in a secluded environment with limited access to distractions (e.g., phones, internet, books), and are led by experienced teachers informed by Buddhist practice and theory; (iii) practitioner-related: meditators who choose to attend intensive retreats might systematically differ from regular meditators who do not attend retreats in personality, intentions, and worldviews; (iv) health behaviour-related: retreatants are commonly encouraged to follow a strict schedule that limits sleep to a number of hours (e.g., wake-up bell at 4am), discourages extensive physical activity (e.g., jogging), and includes a change in diet (e.g., vegan). Notably, out of the 21 studies included in a recent meta-analysis of traditional meditation retreats, none reported the inclusion of measures of particularly unpleasant meditation-related experiences [69]. In brief, we suggest that meditation retreats are an important context for future research on unpleasant meditation-related experiences.

We found no evidence for an association between average session length, session frequency or total lifetime meditation experience and particularly unpleasant meditation-related experiences. Cebolla et al. (2017) [4] reported that unwanted effects were more likely to arise during longer meditation sessions (>40 minutes). Data from Lindahl et al. (2017) [5] indicated a similar trend with more than three quarters of meditators practicing one hour or more per day at the onset of their meditation-related difficulties. Prospective studies that are conducted across a variety of meditation practices and contexts are needed to shed further light on these practice-related relationships.

In addition to the limitations mentioned in the previous paragraphs, several others apply. Although the question concerning particularly unpleasant meditation-related experiences included a subjective causality attribution component, the cross-sectional nature of our data does not allow us to clearly infer whether meditation causally influenced the arising of these experiences. Additionally, only one broad question was used to ask participants about these previous experiences. No additional data on characteristics of the particularly unpleasant meditation-related experiences (e.g., type, severity, duration, level of impairment, ramifications) or the contextual practice-related (e.g., type, intensity, setting) and practitioner-related factors (e.g., conceptual framework, personality, medical history) were collected. Further, including a list of specific examples (i.e., anxiety, fear, distorted emotions or thoughts, altered sense of self or the world) in the question about these experiences may have biased participants’ responses towards recalling these particular experiences over others. Moreover, we collected data over the internet and relied exclusively on self-report. Some of the particularly unpleasant meditation-related experiences might have occurred long before the completion of the survey, whereas the participants’ responses regarding their typical levels of repetitive negative thinking, mindfulness, and self-compassion, might have captured a different period. Relatedly, memory bias could have distorted the retrospective appraisal of these experiences. Most limitations could be addressed by future longitudinal studies employing validated standardised questionnaires (preferably in combination with semi-structured interviews) to offer a more fine-grained analysis of particularly unpleasant meditation-related experiences, their precipitating conditions (including practitioner-related factors such as personality characteristics), and their developmental trajectories. Informed by qualitative and mixed-methods approaches to understanding the variety of contemplative experiences, efforts have been made to validate standardised questionnaires and codebooks assessing the variety of meditation-related experiences (see [5, 70]), which could then be employed in longitudinal research.

### Future directions: Methodological and conceptual considerations

Until recently, only a few overview and opinion papers [71–76] on the potential types of distress associated with meditation in the context of clinical research and practice have been published. This discussion was expanded in the largest critical evaluation of mindfulness and meditation research to date [1], in which 16 of the leading mindfulness researchers proposed a prescriptive research agenda for the “harm, adverse effects, and fallout of meditation practices”. Another important recent contribution was made by Compson (2018) [10], who provided a comprehensive overview of the existing literature, discussed the complexities of contemporary research on “adverse meditation experiences”, and recommended doorways into a mutually enriching dialog around these meditation-related experiences between Buddhist and secular frameworks. However, many issues still need to be resolved. To conclude, we will briefly outline three important issues that have been omitted or not elaborated upon in previous discussions and that need to be addressed in future work.

Firstly, it is unclear how to properly evaluate particularly unpleasant meditation-related experiences. One important issue demanding special attention in meditation research relates to the lack of semantic and conceptual clarity pertaining to these experiences. Characterisations that have been applied to describe these experiences have ranged widely (even within a single article) and have included the following labels: aberrant, adverse, challenging, difficult, distressing, extreme, harmful, impairing, negative, pathological, terrifying, unexpected, and unwanted. This is not a trivial point: operational definitions and conceptualisations are not simply a passive reflection of the researcher’s conceptual framework(s) and epistemological stance (which, in themselves, merit careful explication and analysis); rather, they are also likely to influence meditators, other researchers, study methodology, study outcomes, and their interpretation. This, in turn, will influence the trajectory of this nascent research field along with the perception of funders, policy makers, and the public. To illustrate this point further, research conducted within, for instance, a biomedically informed framework has previously operationalised these experiences as adverse events, side effects, or contraindications (see [1]). While much can be learned from research conducted under the aegis of the biomedical model, the latter cannot be uncritically applied to the study of meditative practices, as it could prevent a more nuanced exploration of unpleasant meditation-related experiences and limit the range of questions that can be posed. For instance, a more integral approach, drawing on findings from various disciplines, might help us elucidate important questions, such as: are particularly unpleasant meditation-related experiences merely negative side effects or rather constitutive, perhaps even essential, elements of (especially rigorous) meditative practice? If the former, why do they occur in the first place and why do some traditional accounts invest them with profound transformative significance? If the latter, how can they be differentiated from phenomenologically similar pathological experiences? Importantly, how does the specific practice context influence the process of distinguishing “normative” progress from “adverse” effects? How are ambiguous meditation-related experiences appraised in different contexts? What role, if any, does the interpretative-conceptual framework adopted by the meditator play in this overarching conciliatory project? Given that many traditional accounts draw a clear distinction between merely negative experiences and experiences that are difficult, but nonetheless integral parts of the meditative practice or essential for receiving its potential benefits, it would obviously be problematic to conflate the two. If essential unpleasant meditation-related experiences are avoided, some of the benefits of the practice could potentially be removed; however, if non-essential unpleasant meditation-related experiences are somehow cultivated this could potentially lead to unnecessary suffering. Importantly, future longitudinal research could offer important insights on whether (and when) unpleasant meditation-related experiences result in positive transformations, in long-term adverse outcomes or in no effects at all. Relatedly, recent discussions have begun to consider how the social dynamics operating within a given practice context might influence the appraisal of “challenging and adverse meditation experiences” and their consequences [77].

Secondly, current discussions on unpleasant meditation-related experiences have not elaborated sufficiently enough on how the empirical research on psychotherapy’s side effects—including the methodological and theoretical challenges encountered in defining, finding, classifying, and assessing side effects—can inform meditation research. Strikingly paralleling meditation research, there is extensive evidence for the positive effects of psychotherapy but a surprising paucity of research on side effects [78]. Another similarity relates to clinical researchers’ recent interest in ameliorating this imbalance in research focus, in addressing the absence of a clear theoretical framework for the definition side effects, and in developing models for the assessment of causality of side effects including treatment fidelity and quality. We propose that the development of a clear procedural model for the conceptualisation, detection, and evaluation of unpleasant, difficult, and distressing meditation-related experiences could be substantially informed by research on the potential side effects of psychotherapy. Recent efforts have been made towards the development of a clear conceptual model of potential harm in mindfulness-based interventions [79].

Thirdly, previous research on unpleasant meditation-related experiences has not clearly distinguished meditation as a means of treating various pathologies from meditation as a means of cultivating well-being (in the traditional sense of *Eudaimonia*, or existential flourishing). Davidson and Dahl (2018) [80] point out that meditation and related contemplative practices were not predominantly developed to treat disease or to have an impact on clinical symptoms. Rather, these practices were intrinsically embedded in soteriological paradigms aimed at human flourishing, radical existential transformation, and awakening. To exemplify these potential differences further, let us consider that mindfulness-based interventions developed for obesity, addiction, depression, anxiety, pain, and a range of other clinical problems, seldom include theoretical and pragmatic frameworks for understanding and cultivating well-being that move beyond a secular, naturalistic (i.e., reductionistic) view of human beings. Conversely, many Buddhist traditions explicate a contemplative path leading to well-defined soteriological goals (e.g., liberation, the end of suffering)–commonly situated in a cosmology that is imbued with a range of religious presuppositions (e.g., rebirth, supranatural powers)–and meditators who do not honour these aspirations but approach the practice primarily for health benefits have previously been discouraged from joining meditation retreats (see [81]). We suggest that future research on unpleasant meditation-related experiences could investigate similarities and differences in the profiles of these experience between these divergent frameworks and varied applications of meditation without reverting to a rhetoric of Buddhist authenticity [82, 83] or assigning some tacit authority to traditional accounts of unpleasant meditation-related experiences *a priori*.

In conclusion, we believe that in order to be able to successfully address the issues briefly outlined here, an open-ended exploration is required, one that would enable us to explicate, broaden, and—if need be—modify the epistemic and methodological frameworks (e.g., medical, psychological, psychotherapeutic, contemplative, spiritual, religious) into which both researchers and meditators are embedded, without giving way to an epistemic asymmetry in which one framework (e.g., neurobiological) explains away another (e.g., phenomenological). We propose that a constructive interdisciplinary discussion of these topics would advance the field and help construct a unified theoretical framework that would do justice to the plurality of traditional and scientific approaches.

## Supporting information

S1 Appendix Questionnaire. (PDF)
