# Supplementary material for: Unpleasant meditation-related experiences in regular meditators: Prevalence, predictors, and conceptual considerations
Source: PLoS One. 2019 May 9;14(5):e0216643. doi: 10.1371/journal.pone.0216643 (PMC6508707; doi:10.1371/journal.pone.0216643)
Supplement: S1 Appendix — (PDF) [file pone.0216643.s001.pdf]

S1 Appendix. Questionnaire.

Schlosser, M., Sparby, T., Vörös, S., Jones, R., Marchant, N.L.

Welcome! Thank you for showing interest in this study. We greatly appreciate the time you dedicate to this project. Please read the following before starting:

The current study aims to further our understanding of the associations between meditation and subjective experiences. To take part you must be at least 18 years old, have a good understanding of the English language, and have a currently ongoing regular meditation practice (at least once a week).

The survey takes 10 minutes to complete. The survey collects information about your meditation experience and asks you to complete several brief questionnaires. We will not record your IP address or any information that could personally identify you. All obtained data are completely anonymous.

The data we collect from this study will help to advance the scientific understanding of meditation practices and their relationship to cognitive and emotional processes. The project has the potential to inform the development of future controlled studies on meditation.

This study has received full ethical approval from UCL's Research Ethics Committee (Project ID: 10043/001). It is up to you to decide whether to take part or not. You are free to withdraw at any time and without giving a reason. You do not have to answer some of the questions if you do not wish to.

By submitting a completed questionnaire you consent to participate.

Note: This survey is optimised for use on laptops/PCs.

Question 1
Please indicate your age.
_____years

Question 2
Please indicate your sex.
- o Male
- o Female

Question 3
What is your current continent of residence?
- o Asia
- o Africa
- o Australia
- o Europe
- o New Zealand
- o North America
- o South America

Question 4
Have you received a university degree (Bachelor's, Master's, PhD)?
- o Yes
- o No

Question 5
Are you religious?
- o Not religious
- o Religious

Question 6
For approximately how long have you been meditating at least once a week?
_____months
_____years

Question 7
Please indicate the type(s) of meditation you practice regularly (on average at least once a week). Choose the categories that best approximate the types of meditation you practice. Please only choose the category 'Other practices' if your regular practice is distinctively different from the listed categories. Please exclude activities such as prayer, yoga, chi gong, and tai chi when reporting your meditation experience.

- **Attentional practices**
  e.g. Mindfulness of breathing/an anapanasati or jhana practice or breath counting or Samatha/samadhi with support, Visualisation or mantra recitation, Kirtan kriya meditation, Choiceless awareness or Samatha/samadhi without support (Tibetan) Transcendental meditation, Mindfulness meditation (e.g., as taught by Mindfulness-Based Stress Reduction programs)
- **Deconstructive practices**
  e.g. Vipassana/insight meditation, Mahamudra (Tibetan), Dzogchen (Tibetan), Shikantaza/'just sitting' (Zen), Self-inquiry (Advaita Vedanta), Koan Practice (Zen)
- **Constructive practices**
  e.g. Lovingkindness meditation/metta or compassion meditation/karuna
- **Other practices**
  (Please specify): __________

Question 8
Since starting your regular meditation practice, how frequently, on average, have you practised meditation?
_____times per week

Question 9
What is the average length of your regular meditation session (in minutes)?
_____minutes

Question 10
Have you ever been on a meditation retreat?
- Yes
- No

Question 11
The Perseverative Thinking Questionnaire (PTQ).
Reference:
Ehring T, Zetsche U, Weidacker K, Wahl K, Schönfeld S, Ehlers A. The Perseverative Thinking Questionnaire (PTQ): Validation of a content-independent measure of repetitive negative thinking. Journal of Behavior Therapy and Experimental Psychiatry. 2011;42(2):225-32.

Question 12
The Self-Compassion Scale (SCS).
Reference:
Neff KD. The Development and Validation of a Scale to Measure Self- Compassion. Self and Identity. 2003;2(3):223-50.

Question 13
The MINDSENS Index.
Reference:
Soler J, Cebolla A, Feliu-Soler A, Demarzo MMP, Pascual JC, Baños R, et al. Relationship between meditative practice and self-reported mindfulness: the MINDSENS composite index. PloS one. 2014;9(1):e86622.

Question 14
Have you ever had any particularly unpleasant experiences (e.g., anxiety, fear, distorted emotions or thoughts, altered sense of self or the world), which you think may have been caused by your meditation practice?
- Yes
- No

Thank you for your participation.
We greatly appreciate the time you dedicated to this project. Your participation helps to further our understanding of the relationship between meditation practices and subjective experiences.
